# When An Apology Implicates A Doctor: Legal Consideration

**DOI:** 10.21315/mjms-04-2025-247

**Published:** 2025-08-30

**Authors:** Choon Yong Alex Tan

**Affiliations:** M. Kandiah Faculty of Medicine and Health Sciences, Universiti Tunku Abdul Rahman, Kajang, Selangor, Malaysia

**Keywords:** apology, liable, disclosure, admission

## Abstract

In recent years, the increasing volume of medical negligence in Malaysia has highlighted crucial concerns regarding the standard of care, informed consent, and liability within the healthcare system. If left unchecked, the rising compensation awards will inevitably affect consumers, namely, the community. Various means exist to potentially prevent litigation, such as engaging in alternative dispute resolution, which includes seeking a sincere apology from the doctor. However, in the judicial system, apologising to patients may also convey the admission of liability, which holds the doctor responsible for negligence. Currently, there is no specific legal framework governing the consequences of apologies in medical error cases. The outcome often depends entirely on the judicial interpretation.

## Introduction

Medical negligence has always been an important issue in Malaysia; it reflects on the dynamic complexities of balancing patient rights and healthcare professional accountability to be governed by an ever-evolving medico-legal framework. In recent years, the increasing volume of medical negligence claims has highlighted important questions regarding the standard of care, informed consent, and liability within the healthcare system.

To date, there is no comprehensive data on the total sum awarded to patients across the nation in 2024 or recently. However, several landmark cases show the increasing compensation trend. In February 2024, the case of Siow Ching Yee v Columbia Asia Sdn Bhd concluded after 14 years, with the private hospital and the anesthesiologist jointly held liable for RM4.5 million (1). In March 2024, the Court of Appeal increased the damages awarded in the case of Nur Adeena Binti Mohd Syahmir v Kerajaan Malaysia from RM5.5 million to RM8.5 million (2, 3). In another case in September 2024, the Court of Appeal awarded RM9.45 million in Syazwani bt Drani v Kerajaan Malaysia, the highest compensation ever awarded to a government hospital, recognising the necessity of long-term supportive medical care (4, 5). These are just some of the notable cases.

If left unchecked, the rising compensation awards will inevitably be exerted on the consumers, consisting of healthcare workers, who face escalating insurance premiums, and patients themselves, who either pay the expenditure out of pocket or rely on insurance schemes (6, 7).

Medical litigation is here to stay, propelled by mounting patient expectations and dissatisfaction. There are several ways to circumvent this route, for instance, by embracing alternative dispute resolutions as an initial intervention or seeking a sincere apology from the doctor.

Academic literature has traditionally stated that the act of apologising to a patient is a courtesy, a countenance of regret for a medical misadventure, more than an admission of negligence (8). Most complaints can be resolved with a genuine apology without actually heading toward court-based litigation. An apology can have a powerful positive impact, promoting therapeutic benefits for patients and encouraging reconciliation in a strained doctor–patient relationship (9).

## The Bane of Apology in Malaysia

The Malaysian Medical Council asserts that doctors have a professional duty when dealing with complaints, and an apology is warranted when deemed appropriate (10). This aligns with the modern clinical practice of respecting the patient’s autonomy, where full disclosure is encouraged.

However, the outcome remains ambiguous when an apology is used to mitigate medical litigation. There is a clear distinction in the nature of the apology. Saying “sorry” in an expression of compassion is more likely not to amount to liability. In contrast, saying “sorry” that implies culpability can at times initiate admissibility in malpractice claims (11).

The case of Norizan bt Abd Rahman v Dr Arthur Samuel was about the plaintiff, who sought treatment for an unwanted pregnancy and poor spacing from the previous pregnancy. The proposed management was a dilatation and curettage, followed immediately by an intrauterine contraceptive device insertion. The right uterine wall was accidentally perforated during the procedure. As such, the only feasible option was hysterectomy. This mishap was subsequently made known to the plaintiff, with an apology from the defendant. The apology became central to the court’s decision, which found the defendant to be negligent.

“… that the defendant and his anesthesiologist admitted to them that they were at fault and had apologised for the mistakes. I am of the opinion that at this juncture the plaintiff’s plea of negligence has been established” (12).

The case of Gurmit Kaur a/p Jaswant Singh v Tung Shin Hospital was also a gynaecological ordeal. The plaintiff was a 38-year-old para 4 diagnosed with a uterine fibroid. She unknowingly consented to a hysterectomy, which she found out postoperatively, and was surprised she could not conceive anymore. The plaintiff alleged that the defendant subsequently informed her that the hysterectomy was performed using the assumption that she did not want to have any more children. The defendant apologised, and the court found that the defendant was negligent in his conduct.

“Upon seeing the plaintiff in a state of shock, the second defendant had apologised to her because he has done hysterectomy on her. The plaintiff’s evidence was not challenged by the second defendant on this point. My view, when the second defendant had apologised to the plaintiff, proves that the second defendant had admitted to a mistake he had done” (13).

In both instances, the apologies offered by these two independent doctors in two different scenarios were interpreted as admissions of guilt, thereby entailing liability in the eyes of the court. These cases could show how the act of apologising, once regarded as pure and innocent, can unintentionally be used to incriminate doctors.

A further concern arises when medical indemnity policies exclude coverage, invoking the “assumed liability” clause (14). If an apology creates a perception of fault, the insurer may technically deny any coverage if the doctor has assumed liability outside the policy scope.

In a non-medical context, in the case of Mammoth Empire Construction Sdn Bhd v Lifomax Woodbuild Sdn Bhd, the Court of Appeal considered a letter of apology as an element of the admission of liability (15). Although this case has not yet been cited in medical litigation, it serves as a cautionary example.

## Moving Forward

The question to ask is not “How should a doctor apologise?” but rather “Should a doctor apologise?” Without a blanket of protection in Malaysia, apologising could lead to an unpropitious legal outcome. Some countries have enacted apology laws that provide legal protection for doctors offering sincere apologies, even when these include indirect admissions of fault (16).

Apology laws not only mandate and permit doctors to openly apologise for medical errors despite full disclosure but also grant them immunity against legal liability (17); this means that the entire apology, in addition to any indirect admission of fault, is inadmissible during the trial.

The relevance of such protection is undeniable owing to the medico-legal ambiguity tethering apologies. It is fitting to have a structured legal approach to manage medical errors, with emphasis on ethical obligations through regulatory reforms, where transparency underscores the very essence of disclosure (18). Well-meaning apologies should not be a foundation for debate.

## Conclusion

Enacting apology laws in Malaysia is not just another legal reform; it is a step forward, where apology is an ethical and cultural value in Malaysian Asians (19). This country lacks a definite legal framework regarding apologies for medical errors. The outcome solely relies on judicial interpretation. It is time for Malaysia to address the negative legal repercussions associated with an apology.
